# ADVANCE CARE PLANNING ASSIGNMENTS IN UNDERGRADUATE COURSES: EXAMINING STRESS APPRAISALS, COPING, AND RESPONSES

**DOI:** 10.1093/geroni/igz038.1975

**Published:** 2019-11-08

**Authors:** Maureen Templeman, Victoria R Marino, Debra J Dobbs, William E Haley

**Affiliations:** University of South Florida, Tampa, Florida, United States

## Abstract

A common assignment in undergraduate aging courses requires students to consider or complete advance care plans for themselves. Due to the sensitive nature of this topic, it is important to consider student appraisals of such assignments. Using the transactional model of stress and coping, the current study used content analysis to examine student reactions to considering their own advance care planning. Participants included 151 students, diverse in race/ethnicity, in a Psychology of Aging undergraduate course at a large public university in the United States. Students were first exposed to course material related to death, dying, and bereavement, in addition to detailed advance directive forms, and were subsequently asked to discuss the importance of, and their preparedness for, their own advance care planning via a written assignment. Codes represented primary and secondary appraisals, coping efforts, and responses, and data was analyzed using ATLAS.ti software. Findings suggest that primary appraisals (perception of effect on well-being) ranged from positive to threatening, and secondary appraisals (perception of coping resources) focused on availability of internal and/or external resources. Themes such as traumatic experiences with death, feeling ill-prepared due to lack of knowledge or experience, and welcoming the opportunity to consider completing advance care plans emerged. Overall, results suggest that when designing assignments that require completion of advance care plans among undergraduate students, students should have the opportunity to discuss personal concerns first, and that some initial class activities should focus on preparatory tasks rather than just completion of advance care plans.

